# Innovation of strip fertilization planting for rice straw crushing with back-throwing and interrow-laying

**DOI:** 10.1186/s13007-022-00862-6

**Published:** 2022-03-15

**Authors:** Yinyan Shi, Ye Jiang, Xiaochan Wang, Haiming Yu, Hui Liu, Jingbo Chen

**Affiliations:** 1 Department of Electrical Engineering, College of Engineering, Nanjing Agricultural University, Box 96, 40 Dianjiangtai Road, Pukou, Nanjing, 210031 China; 2 Kunshan Station for Agricultural Mechanization Technology Extension, Kunshan, 215300 China

**Keywords:** Straw returning, Compound operation, Clean-area planting, Inter-row stacking, Strip seeding

## Abstract

**Background:**

In order to improve the current situations in rice–wheat rotation region in the middle and lower reaches of the Yangtze River, such as large amount of rice straw, complex returning process, short-time stubble connection, high power consumption, poor smoothness and especially unstable performance, and further promote the resource utilization process of full straw returning in Jiangsu province, this study, combined with the agronomic requirements of wheat sowing in rice-stubble land, developed an innovation of strip fertilization planting for straw crushing with back-throwing and interrow-laying in full stubble fields.

**Results:**

Structural design and theoretical analysis were carried out on key components such as straw crushing device, broken-straw control device, soil rotary-tillage device and power transmission device, etc., to determine the corresponding structure and operating performance parameters, and then the field performance and verification tests were completed on the uniformity of inter-row mulching-straw *Y*_*1*_ and the variability of seed-band width *Y*_*2*_. The results showed that the crushing spindle rotation-speed *A* had an extremely significant impact on *Y*_*1*_, followed by the machine ground speed *B*. The conveying impeller rotation-speed *C* had an extremely significant effect on *Y*_*2*_, also secondary to the machine ground speed *B*. And the superior combination of factor levels as *A*_*2*_*B*_*2*_*C*_*2*_ was adopted through the comprehensive power energy consumption analysis. The verification test results indicated that under the optimized operation parameter combination, namely, when the crushing spindle rotation-speed *A* was 2100 r/min, the machine ground speed *B* was 0.8 m/s, and the conveying impeller rotation-speed *C* was 210 r/min, the mean value of inter-row straw uniformity *Y*_*1*_ and seed-band width variation *Y*_*2*_ were 90.85% and 10.73%, respectively, after machine operation.

**Conclusion:**

It meets the requirements of operation quality and planting agronomy of relevant protective tillage machinery, and provides technical and equipment support for the research and development of similar straw crushing and no-tillage sowing.

## Background

Avoiding burning and polluting the environment, straw crushing and returning to the field increases soil organic matter, improves soil structure, promotes microbial activity and the development of crop roots, and plays a positive role in increasing fertility and yield [1, 2]. The no-till and less-till sowing technology as a normal operation method for implementing the concept of conservation tillage, it focuses on the main control target of protecting the ecological environment of the cultivated land, reducing the number of operation equipment landed the field, decreasing soil erosion and structural damage, and preventing soil desertification and compaction. It is the current important production mode in the main grain production areas in the middle and lower reaches of the Yangtze River to improve production efficiency and economic benefits [3].

As a typical rice–wheat rotation area in Jiangsu Province, the large amount of rice stubble is particularly prominent in the large-scale planting process, which hinders the smooth operation of fertilization and planting machines, destroys the good growth environment of crops, and restricts the strong promotion and application of no-tillage planting technology [4], especially the lack of straw crushing-returning technology and equipment that can meet the local agronomic requirements, has seriously affected the mechanization development of overall process for rice production and the safety of food production [5, 6]. Therefore, it is particularly urgent to develop machinery and equipment for returning straw to the field and planting with less tillage and no-tillage suitable for the rice–wheat rotation system.

In recent years, as the country continues to advocate the comprehensive utilization of straw resources and the advancement of land rotation and fallow strategies, more and more scholars at home and abroad have paid attention to the research and development of key technologies and equipment for straw crushing and no-tillage fertilization sowing [7–9]. ELFATIH [10] et al., in order to realize the reuse of crop residue as compost material, optimized and improved the feed-in straw crushing device designed to improve the straw crushing efficiency and productivity. Liu Peng [11, 12] and other researches proposed a method of crushing and returning corn stalks to the field in the form of different speed rollers and dynamic double supports. Through the design of pick-up and crushing blades and roller sliding support blades, the width and uniformity of broken straw scattering can be adjusted, and the pass rate of corn stalk crushing after operation is 92.58%. The 1JHL-2 straw deep burying and returning machine developed by Tian Yang et al. [13] can collect two ridges of straw and bury them in a ditch, so as to realize alternate deep burying of straw in ridge and ditch. SIDHU [14] and others developed a suitable 9-row turbocharged wheat no-tillage planter with straw crushing and returning to the field in rice stubble fields, which reduced fuel consumption costs, optimized the optimal planting period, and promoted direct wheat production in rice stubble fields. The 2BMFJ series no-tillage precision planter designed by Chen Haitao [15] used a lateral throwing method to collect the stubble on the side of the planting area, which solved the problems of difficult planting and slow rise of low temperature in the later period. Wang Weiwei et al. [16] designed an active straw shifting anti-blocking device to ensure the passability of the corn no-tillage planter when the wheat stubble is high and the straw is fully returned to the field. Throughout the current stage, the research on crop planting technology and equipment under the condition of straw mulching mainly adopts methods such as crushing and throwing mulching, ploughing and burying, mixed burying in soil, ditching and deep burying, and collecting straw on the side to accelerate the decomposition of straw in the field [17–20]. However, there are few researches on the compound operation technology of crushing all straws in the field and collecting mulching between rows and sowing in the clean area [21–23]. In particular, there are few reports on the stability of operation quality, such as the uniformity of ridge pattern and the variation of seed band width.

Therefore, in view of the special planting environment of tight Crops for Rotation under the condition of full straw return in the rice–wheat rotation area in the middle and lower reaches of the Yangtze River, this study continued the technical concept of strip fertilization and seeding in "clean area" (no straw obstacle) [24–26], and developed an innovation of strip fertilization planting for straw crushing with back-throwing and interrow-laying. In a single operation, multiple production processes such as straw crushing, broken straw gathering and mulching, seed-bed arrangement, fertilization and sowing in clean area can be realized, which not only guaranteed the farming time under the rice–wheat rotation, but also realized the high-quality and smooth no-tillage planting operation of the full amount of straw land. In addition, it can ensure the thermal insulation and moisture retention of the straw-covered ground surface, and the full contact between the seeds and the soil, and the phenomenon of difficult seedling emergence, weak seedlings and dead seedlings caused by straw mulching can be avoided. It would provide new technology and equipment for the realization of straw-free and high-quality machine sowing wheat operations in rice stubble fields, and provide new ideas for the development of conservation tillage concepts.

## Methods

### Planter structure

**Fig. 1:**
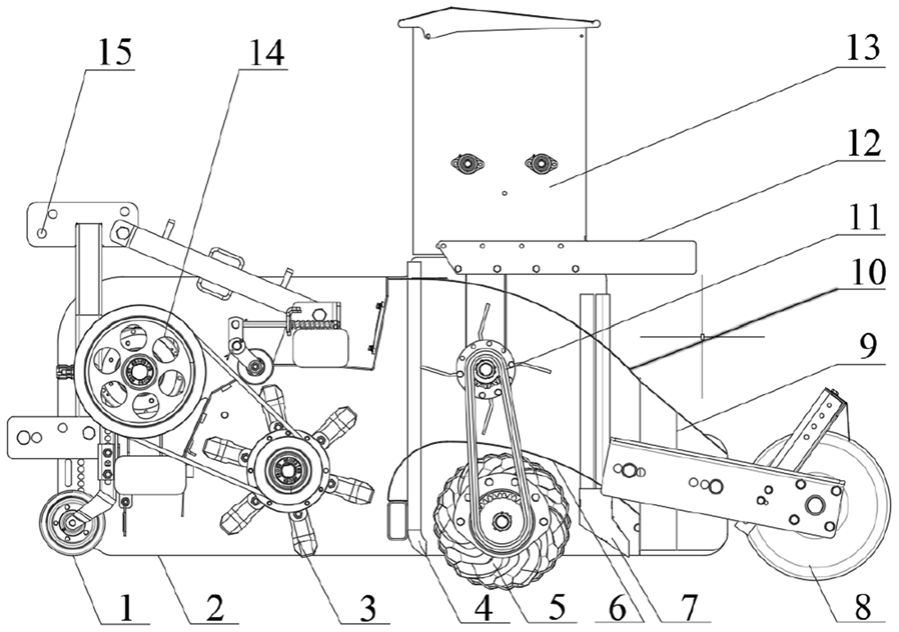
Structural diagram of the strip fertilization planter for straw crushing. 1. Depth limiting device; 2. Rack; 3. Crushing device; 4. Fertilizer falling mouth; 5. Rotary tillage device; 6. Cavity bottom plate; 7. Seed metering opening; 8. Soil suppression device; 9. Diversion device; 10. Cavity cover plate; 11. Throwing blower; 12. Bench; 13. Fertilizing and sowing device; 14. Transmission system; 15. Suspension system

Figure 1 illustrates the overall structure of the strip fertilization planter for straw crushing with back-throwing and interrow-laying, it is mainly composed of a rack, a suspension device, a transmission system, a straw crushing device, a broken-straw control device (post-throwing conveying device, diversion spreading device), a rotary tillage and soil covering device, and a strip fertilizing and seeding device. And the main technical parameters are shown in Table 1.

**Table 1:** Technical parameters of the machine

Parameter	Value
Dimensions (length × width × height) /(mm × mm × mm)	2500 × 2300 × 1400
Weight/kg	960
Matched power/kW	≥ 70
Working width/mm	2200
Crushing spindle speed/(r min^−1^)	1850–2300
Rotary-tillage spindle speed/(r min^−1^)	200–300
Throwing impeller speed/(r min^−1^)	180–250
Straw-mulching rows	6
Straw-mulching width/mm	210
Seed-band rows	5
Seed-band width/mm	185
Working speed/(m s^−1^)	0.8–1.5
Working efficiency /(hm^2^ h^−1^)	0.65–1.1

### Working principle

The developed strip fertilization planter adopted a three-point suspension connection. The output power of the tractor was transmitted to the left and right sides of the planter through a gearbox, by the gear transmission matched with the chain drive on the left side of the rack, the power of the impeller shaft for straw throwing was driven. The right side of the impeller shaft provided power for the rotary tiller shaft through a gear and chain drive, and the power for the straw crushing shaft was provided through a belt drive at the right side of the rack.

When working in the field, the sufficient driving for the whole machine was provided by the tractor connected to the power input of the gearbox at a standard speed of 720 r/min, and the matching required power to the crushing spindle and the rotary spindle was transmitted through the multi-stage wedge-belt. The full coverage straw on the ground surface smoothly entered into the crushing cavity under the pressure of a depth-limiting roller, and the high-speed and counter-rotating crushing movable blades picked up the straw within the working width with the help of centrifugal inertial airflow, then cooperating with the array of fixed blades on the top of the cavity, the straw was crushed. Then the broken straw entered with the high-speed airflow of the rotating blades, was captured by the straw throwing device (throwing impeller) in the cavity, and then accelerated to the rear spraying again. After crossing the soil rotary tillage device, under the reasonable guidance of the broken-straw regulating device, these broken straw were paved on both sides of the ground wheel in the non-sown region, forming 5 seeding belts without straw obstacles and 6 straw covering areas evenly spread between rows. A temporary straw-free cleaning zone was formed between the crushing device and the diversion device, where the rotary tillage, soil crushing, band-shaped fertilization and seeding operations were completed, avoiding the interference of straw on the soil rotary tillage and the seed bed arrangement, and creating a fertilization and seeding condition without the disturbance of straw barriers. Meanwhile, the necessary soil-covering suppression process would be completed subsequently, and finally a high-quality and smooth less-tillage sowing operations with full straw crushing and strip covering in rice stubble fields would be achieved.

### Design of straw crushing device

The straw crushing device is mainly composed of a blade spindle, a blade holder, a crushing blade group, a fixed blade group and other parts, and its purpose is to pick up the rice straw and stubble on the ground surface and return them to the field after crushing. In order to achieve the ideal straw picking and crushing effect, it is necessary for the crushing blades to be correctly selected and rationally arranged, and the blade spindle to meet a certain speed, so that the blade tip has a sufficiently large linear velocity, and at the same time avoids violent vibration [27]. The configuration design of the crushing fixed blade needs to match with the crushing swing blade, so that the straw is crushed into sections or fibers of qualified length, and a uniform straw flow is formed and sprayed to the rear of the broken-straw conveying cavity.

### Structure design of crushing blade

**Fig. 2:**
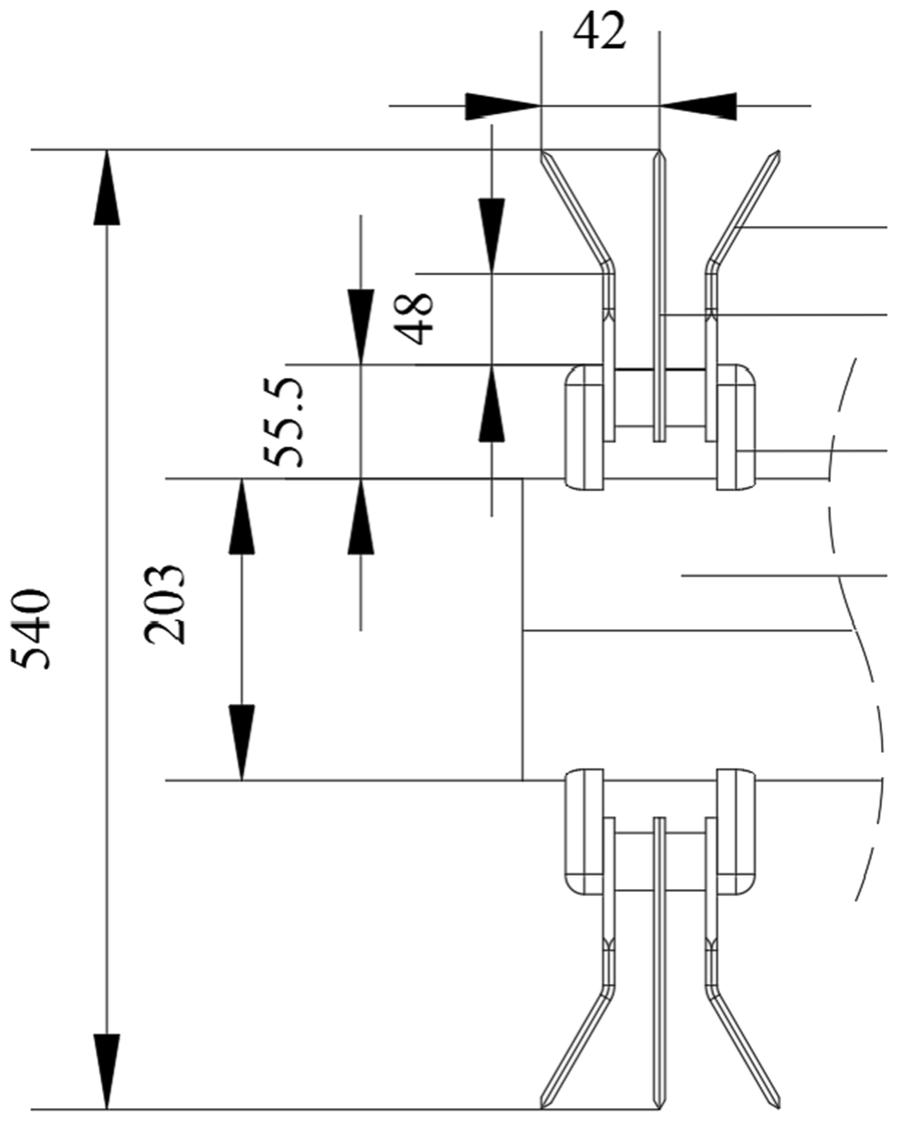
Structural diagram of the crushing blades

Refer to the design experience of the research group on straw crushing devices [21, 24], taking into account the strong crushing ability of the straight blade and the better picking effect of the L-shaped blade, combined with the agronomic requirements of wheat planting in the rice–wheat rotation area and the design requirements of this strip fertilization planter, a straight blade and two L-shaped blades are selected to form a combined swing blade (as shown in Fig. 2), and the blades are positioned by sleeves to ensure that the blade set can be flexibly flung and cut while avoiding axial shaking and mutual interference. The main design parameters are: 160 mm × 60 mm × 6 mm (length × width × thickness), the bending angle is 120°, the cutting edge angle is 25°, and the material is 65Mn steel after quenching treatment to improve its strength, hardness and certain wear resistance, as well as to improve the straw crushing effect and the service life of the swing blades.

In order to be able to work in coordination with the broken straw conveying mechanism, the traditional L-shaped cutter was improved, and the sharp corners at both ends of the scimitar are removed, so that the broken straw can be smoothly thrown out of the surface of the crushing cutter, and then can be smoothly captured by the straw throwing device. This new cutter has both the crushing ability of the traditional straight blade and the pick-up capacity of the L-shaped blade. It has good symmetry, avoids the unbalanced inertia and the vibration of the whole machine, and can meet the working requirements of the straw crushing device and the broken-straw conveying mechanism.

### Blades arrangement and spindle design

**Fig. 3:**
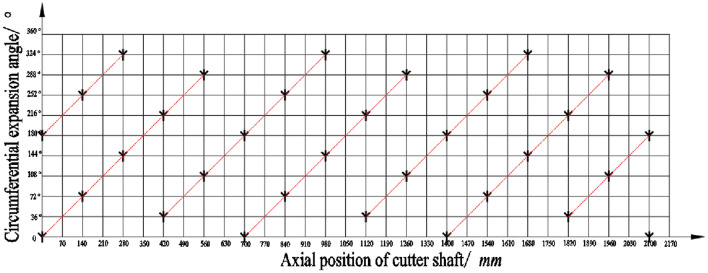
Expansion drawing of chopping blades arrangement

According to design experience, an appropriate increase in the number of movable blades can help improve the straw picking rate and crushing effect, but an excessive number of rotating blades can easily cause vibration of the whole machine and increase the power consumption of the planter. Refer to the same analysis method in "Agricultural Machinery Design Manual" and the literature [24], the arrangement of the movable blade set adopts the common double-helix staggered symmetry method, and is mounted on the blade seat welded on the surface of the blade roll through the pin hinge. The phase angle of the double helix is 180°, the distance between the adjacent blade groups on each helix is 140 mm, and the circumferential interval is 72°, so as to ensure that the working area of the cutter is partially overlaps, and avoid missed picking and incomplete crushing. Because the effective working width of the whole machine is 2200 mm, a total of 32 movable blade sets are calculated according to this arrangement, and Fig. 3 shows the specific arrangement.

In order to improve the straw picking and crushing performance of the strip fertilization planter, the crushing blade spindle was designed to rotate in the opposite direction (reverse), and the instantaneous support sliding cutting mode was formed between the movable blade group and the fixed blade group, which puts forward certain requirements for the cutting speed of the movable blades (that is the rotation speed of the cutter spindle). The linear velocity of the crushing blade tip is a key parameter that affects the quality of straw crushing. Too high speed will increase the unbalance factor of the cutter roller itself, which is easy to cause vibration and increase power consumption. According to the working parameters of the existing machines [24], the linear velocity *v*_*g*_ of the swing blade tip should meet 34–48 m/s, and the rotation radius *r*_*f*_ of the cutter spindle should meet 240-350 mm. Here, the design in this study *v*_*g*_ = 40 m/s, *r*_*f*_ = 270 mm. Combined with the requirements of the sowing speed in the region and the picking demand of the swing cutter on the ground straw, and comprehensively considering the field surface unevenness and topographic undulations, the designed planter forward speed *v* = 1 m/s and the ground distance of the cutter tip determined by the depth-limiting device was selected *h*_*t*_ = 90 mm. According to the following empirical calculation formula of the cutter spindle speed *n*:

1 $$n \ge \frac{{30\left( {v_{g} - v} \right)}}{{\pi \left( {r_{f} - t} \right)}}$$

The cutter spindle speed *n* ≥ 2070r/min was calculated. From the perspective of power balance and power consumption, on the premise of ensuring the straw crushing effect, the spindle rotation speed of the blades should be reduced as much as possible. Therefore, the rotation speed of the crushing spindle *n* = 2100r/min was designed in this study.

### Design of the regulating control device for broken straw

The framework structure of the regulating control device for broken straw is shown in Fig. 1, which is mainly composed of a post-throwing conveying device, a diversion strip-paving device, and a closed cavity for the broken straw flow formed by a bottom plate and a cover plate. During the working process, the picked up and crushed straws were thrown into the flow cavity under the action of inertial airflow, and then captured by the post-throwing conveying device that rotated in the opposite direction at a certain speed. Under the action of the rotating kinetic energy of the throwing impeller, the broken straw was further thrown backward to the distributary strip paving device, then flowed to the outlet under the action of the airflow in the cavity, and were distributed by the guide device to both sides for strip stacking and forming a ridge, which collected and covered the non-sown strip-shaped area between the adjacent guide plates, forming a regular inter-row covered-straw belt (between rows).

### Post-throwing conveying device

In order to achieve the smooth conveying effect for broken straw, the post-throwing conveying device must be highly coupled with the straw picking and crushing device. During the working process, the amount of straw thrown by the conveying device should be greater than or equal to its maximum feeding amount, so as to meet the operating efficiency and capture all the straw ejected from the straw crushing device [28]. At the same time, the throwing blades should provide sufficient initial speed for the broken straw to ensure that the straw can be efficiently thrown to the preset diversion strip-paving device. Therefore, in order to ensure that the broken straw captured by the conveying device can be thrown backward smoothly and avoid the accumulation and blockage of the broken straw in the conveying cavity, the input and output volumetric flow rate (t/h) of the broken straw per unit time should meet the following relationship:

2 $$3600 \cdot v_{d} \cdot S_{d} \cdot \rho \ge 3600 \cdot q \cdot v \cdot W$$

where, *v*_*d*_ is the straw throwing speed, m/s; *S*_*d*_ is the cross-sectional area of the throwing outlet, m^2^; *ρ* is the straw bulk density, t/m^3^; *q* is the straw amount per unit area, t/m^2^; *v* is the machine forward operating speed, m/s; *W* is the working width, m.

**Fig.4:**
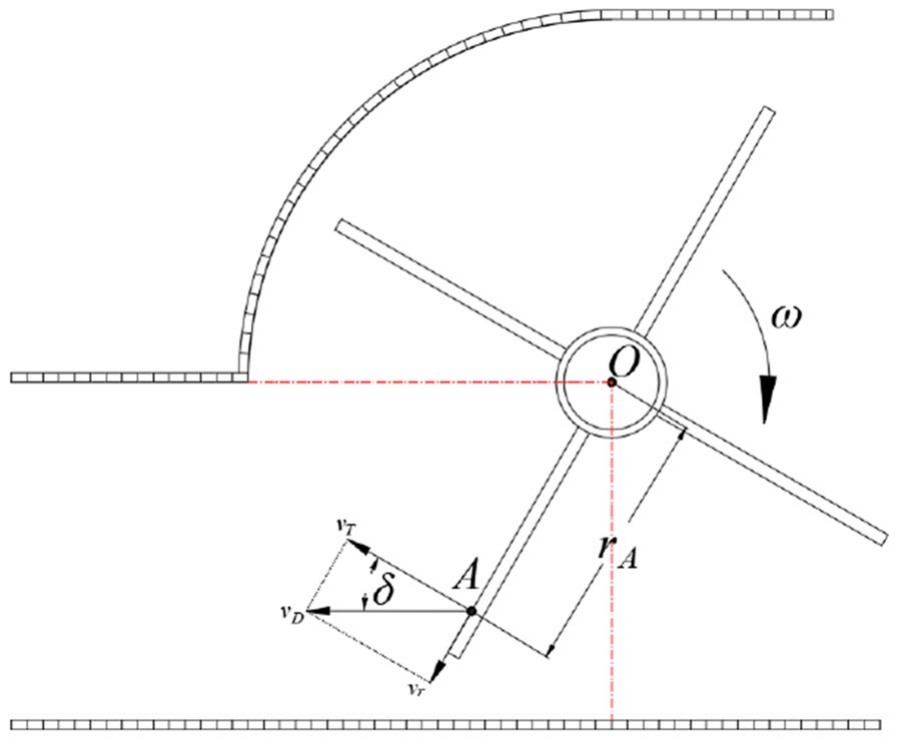
Motion analysis of broken-straw throwing process

During the post-throwing conveying process, the broken straw will be produced an implicative velocity with the rotation of the impeller, a relative velocity along the radial direction of the impeller vane will be generated under the action of centrifugal force, and the direction of the combined velocity is the direction in which the broken straw is thrown [29]. The composite motion analysis generated in the conveying process of the broken straw is shown in Fig. 4, the broken straw follows the throwing impeller to rotate around the axis at a constant angular velocity *ω* (uniform circumferential motion), and the initial throwing velocity *v*_*d*_ of any broken straw *A* is the compound velocity between the tangential velocity *v*_*T*_ affected by the impeller vane and the radial velocity *v*_*r*_ under the action of the inertia effect, then there is.

3 $$\left\{ \begin{gathered} v_{d} = \frac{{v_{T} }}{\cos \delta } \hfill \\ v_{T} = \omega r_{A} = \frac{2\pi n}{{60}}r_{A} = \frac{{\pi nr_{A} }}{30} \hfill \\ \end{gathered} \right.$$

where, *δ* is the included angle between the broken-straw throwing velocity and the tangential velocity, °; *r*_*A*_ is the gyration radius of the broken straw *A* (distance from the rotation axis of *O*), m); *n* is the rotate speed of rotary impeller spindle, r/min.

Combined with the Eqs. (2) and (3), it can be calculated that the necessary conditions to ensure the smooth post-throwing and conveying for the broken straw are as follows:

4 $$n \ge \frac{30qvW\cos \beta }{{\pi r_{A} S_{d} \rho }}$$

With reference to relevant industry standards and literatures [24, 29], the rice straw bulk density *ρ* = 0.045t/m^3^, and the field straw coverage is 1.45 kg/m^2^, that is, *q* = 1.45 kg/m^2^. According to the design dimensions of the whole machine, the rotary radius of the throwing impeller is 300 mm. In order to simplify the calculation, the average straw-discharge effect of the conveying device is evaluated by the throwing velocity of the broken straw at the midpoint of the impeller vane, that is, *r*_*A*_ = 150 mm. The designed working width of the whole machine *W* = 2200 mm, and the discharging outlet area of the conveying cavity for the crushed straw *S*_*d*_ = 2200 × 600 mm = 1.32m^2^. Generally, the field walking speed of the fertilizing and seeding machine *v* = 1 m/s. Therefore, according to formula (4), it can be calculated that the rotate speed of the throwing impeller *n* ≥ 205.13 r/min, and *n* = 210 r/min after rounding.

### Diversion strip-paving device

**Fig.5:**
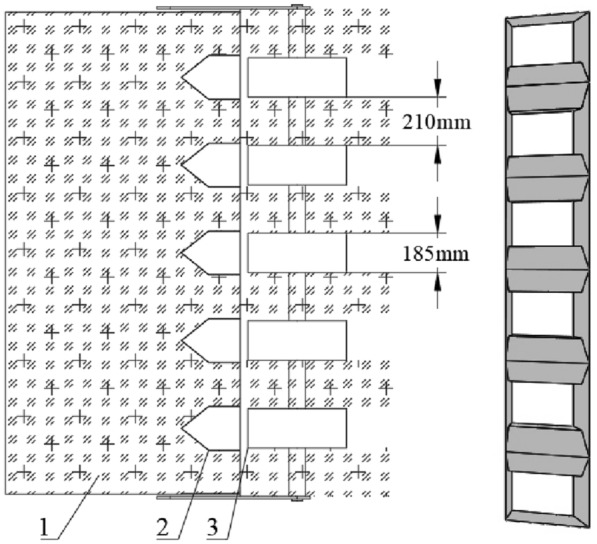
Schematic diagram of straw strip-laying device. 1. Full amount of straw; 2. Diversion strip-paving device; 3. Clean-area sowing belt

As a key component to complete the interrow collecting and mulching process for broken straw, the diversion strip-paving device is also the basic condition for realizing the fertilization and seeding operation in the clean area, its structure will directly affect the effect of the straw collection mulching and the quality of the clean-area seed belt. The working process of the straw diversion strip-paving device as shown in Fig. 5, the broken straw thrown backwards by the rotating impeller vane, are blocked by 5 sets of fixed array adjusting control devices at the exit of the conveying cavity and then diverted along both sides of the deflector. The straw flow slides down and is laid on the ground to form 6 regular straw mulching belts, and 5 clean-area sowing belts corresponding to the diversion control device are formed between adjacent straw covering belts.

Referring to the agronomic requirements of wheat planting in the test area (rice–wheat rotation area in the middle and lower reaches of the Yangtze River) [24], the transverse width of the deflector plate is designed to be 185 mm, and the adjacent diversion strip-paving devices are spaced 210 mm apart and evenly distributed along the width, they are fixed between the cover plate and the bottom plate at the outlet of the conveying cavity by bolts. According to the relevant design experience of the research team’s previous research, discretize the movement process of broken straw particles on the diversion slope at a certain time *t*, establish the instantaneous motion differential equation of the broken straw, and analyze the corresponding law of motion. The specific force balance equation and the dynamics analysis procedure are as seen the literature [21, 23, 24, 26], so we won’t repeat it here. Therefore, the half outward expansion angle of the diversion device designed in this study is 45°, the sliding-push curve of the center blade edge is a parabolic segment, the effective diversion length is 120 mm, the diversion height is 250 mm, and the installation clearance is 20 mm, which can meet the requirements of the strip-paving operation for broken straw.

### Design of the rotary tillage and soil covering device

**Fig.6:**
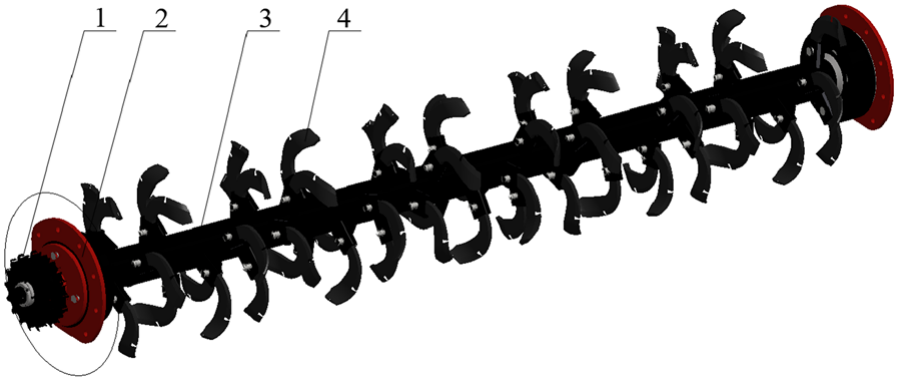
Structural diagram of rotary tillage and soil covering device. 1. Transmission gear; 2. Fixed side plate; 3. Rotary-tillage cutter spindle; 3. Rotary-tillage cutter sets

The rotary tillage and soil covering device arranged the seed beds in the clean area without straw obstacle formed under the cavity bottom plate, which avoided the trouble of the large amount of straw residue jamming and winding, improved the inconsistent sowing depth caused by the unflatness of the paddy planting field, and enhanced the passability and operation quality of the planter. The specific structure of the developed device is shown in Fig. 6, in order to reduce the power loss caused by the unbalanced inertia of the rotary-tillage spindle during the soil rotary tillage process, the rotary tillage cutter set were designed to be arranged in a full-width spiral pattern within the operating range, and the cutter spindle is in shallow rotation in the forward positive direction. Combined with the overall structure of the developed planter and the requirements of wheat sowing depth in the regional rice stubble field [21, 22], the national standard IT195 rotary tillage scimitar was selected, and the tilling depth was adjustable between 60 and 100 mm. At the same time, in order to reduce the weight of the whole machine, a hollow steel pipe with a diameter of 80 mm and a wall thickness of 4 mm was selected for the rotary-tillage cutter spindle.

### Determination of rotary tillage parameters

In the rotary tillage process, the rotation speed of the cutter spindle directly affects the quality of soil crushing and the effect of seed bed finishing, and a reasonable rotation speed of the cutter spindle can also reduce the overall power consumption of the rotary tillage device. Its absolute motion is a compound motion of walking linear motion and self-rotating motion, the specific process analysis is similar to the motion of cutting and crushing blade set in the previous studies (not further repeated here) [24, 26]. When the rotary-tillage cutter roller rotates at angular velocity *ω* forward and the whole machine advances at velocity *v*, the motion equation of any point *B*(*x*, *y*) on the lateral cutting edge of the rotary tiller blade in unit time *t* can be expressed as:

5 $$\left\{ \begin{gathered} \left\{ \begin{gathered} x = vt + Rcos(\omega t) \hfill \\ y = Rsin(\omega t) \hfill \\ v_{x} = x^{\prime} = \frac{dx}{{dt}} = v - \omega Rsin(\omega t) \hfill \\ v_{y} = y^{\prime} = \frac{dy}{{dt}} = \omega Rcos(\omega t) \hfill \\ \end{gathered} \right. \hfill \\ v_{b} = \sqrt {v_{x}^{2} + v_{y}^{2} } = \sqrt {v^{2} - 2v\omega Rsin(\omega t) + \left( {\omega R} \right)^{2} } \hfill \\ \end{gathered} \right.$$

where, the forward direction of the whole machine traction is the positive direction of the *x*-axis, and the vertical forward direction upwards is the positive direction of the *y*-axis; *ω* is the rotational angular velocity of the rotary-tillage cutter spindle, rad/s; *R* is the rotary tiller radius of gyration, m; *v* is the planter forward speed, m/s; *v*_*x*_ is the sub-velocity of any point *B* on the rotary-tillage blade in the *x* direction, m/s; *v*_*y*_ is the sub-velocity of any point *B* on the rotary-tillage blade in the *y* direction, m/s; *v*_*b*_ is the absolute movement velocity of any point *B* on the rotary-tillage blade, m/s.

According to the literature [21], it can be known that the linear speed of the blade tip of the soil rotary tillage device is greater than 5 m/s to achieve a good stubble breaking and shallow rotary-tillage effect. Substituting into Eq. (5), *ω* > 25.12 rad/s can be calculated, that is, the rotation speed of the rotary-tillage cutter spindle > 240r/min, in order to reduce unnecessary power consumption and consider the design margin, take the cutter spindle speed = 250r/min.

### Analysis of soil covering process

**Fig. 7:**
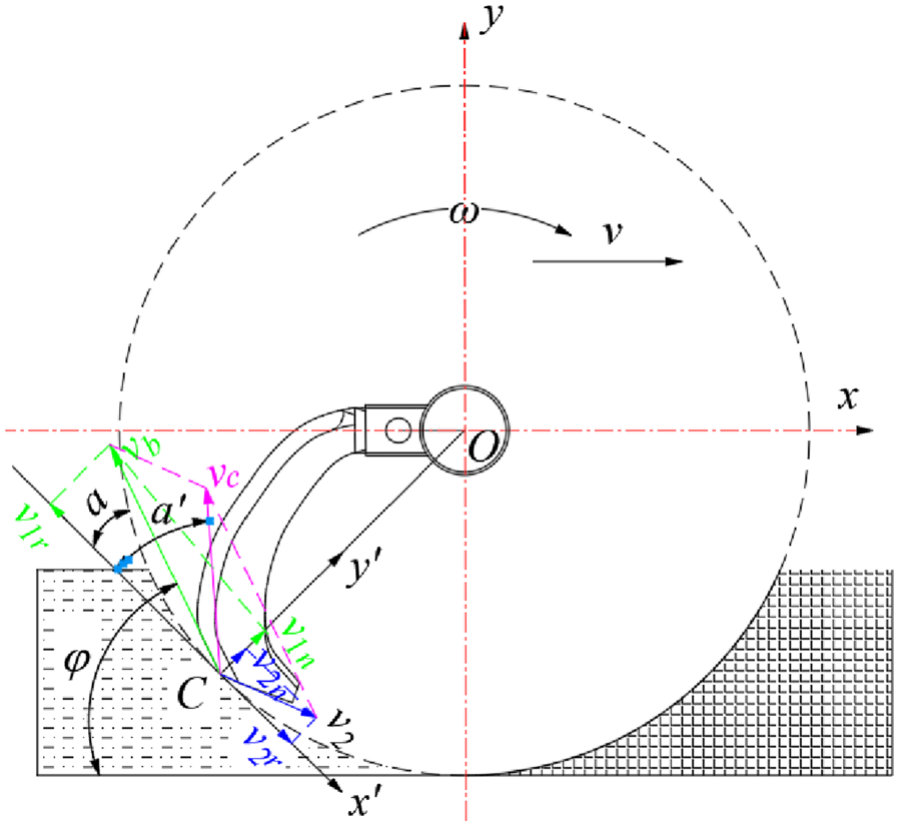
Motion analysis of crushed soil particles

The rotary tillage and soil covering device uses the crushed soil thrown by the rotary-tillage cutter to cover the seed and fertilizer in layers and spatial positioning, so the soil throwing performance of the rotary-tillage blades plays a key role in the effect of soil covering after sowing [27]. Therefore, it is necessary to analyze the motion characteristics of the broken soil particles thrown by rotary tillage. Similarly, the forward direction of the planter is the positive direction of the *x*-axis, the upward direction of the vertical ground is the positive direction of the *y*-axis, and the rotation center of the rotary tiller axis is the origin *O*. The coordinate system *xOy* as shown in Fig. 7 is established.

In the process of soil crushing, considering the impact between the tangent plane of the rotary tillage cutter and the soil [30], when the rotary tiller cuts the soil particle *C* at a certain cutting angle *α* and linear velocity *v*_*b*_, a velocity coordinate *x’Cy’* with *C* as the origin is established. Then the proportional relationship between the normal and tangential components of the relative velocity of soil particle *C* in the dynamic coordinate system *x’Cy’* before and after the impact can be expressed as:

6 $$\left\{ \begin{gathered} \left\{ \begin{gathered} \frac{{v_{2n} }}{{v_{1n} }} = \frac{{v_{2n} }}{{v_{b} \sin \alpha }} = k \hfill \\ \frac{{v_{2r} }}{{v_{1r} }} = \frac{{v_{2r} }}{{v_{b} \cos \alpha }} = 1 - f \hfill \\ \end{gathered} \right. \hfill \\ \alpha = \varphi + 90^{^\circ } - \omega t \hfill \\ \end{gathered} \right.$$

where, *v*_*1n*_ is the normal sub-velocity of the rotary tiller, m/s; *v*_*2n*_ is the normal sub-velocity of the soil particles after collision, m/s; *v*_*1r*_ is the tangential sub-velocity of the rotary tiller, m/s; *v*_*2r*_ is the tangential sub-velocity of the soil particles after collision, m/s; *k* is the recovery coefficient of soil particle collision, generally taken as 0.4; *f* is the instantaneous friction coefficient of soil particles, generally taken as 0.5; *φ* is the angle between the absolute movement velocity of the rotary tiller *v*_*b*_ and the *x*-axis, °; *α* is the angle between the absolute movement velocity of the rotary tiller *v*_*b*_ and the *x’* axis, °.

And the absolute velocity equation of soil particle *C* can be expressed as:

7 $$\left\{ \begin{gathered} v_{c} = \sqrt {(v_{1r} - v_{2r} )^{2} + (v_{1n} + v_{2n} )^{2} } = v_{b} \sqrt {\left[ {(1 + k)\sin \alpha } \right]^{2} + (f\cos \alpha )^{2} } \hfill \\ \tan \alpha ^{\prime} = \frac{{v_{1n} + v_{2n} }}{{v_{1r} - v_{2r} }} = \frac{1 + k}{f}\tan \alpha \Rightarrow \alpha ^{\prime} = \arctan \left( {\frac{1 + k}{f}\tan \alpha } \right) \hfill \\ \end{gathered} \right.$$

where, *v*_*c*_ is the absolute velocity of soil particle *C* after collision, m/s; *α'* is the angle between the absolute velocity *v*_*c*_ of the soil particle C and the *x’* axis after the collision, °.

If the air resistance was not taken into account, the particle *C* was idealized as an independent discrete body, neglecting the interaction with the surrounding soil, the continuity equation of the motion trajectory of the soil particle *C*, the first derivative with respect to time *t* can be expressed as:

8 $$\left\{ \begin{gathered} \begin{array}{*{20}l} {x_{c} = x_{0} { + }v_{c} t\cos {(}\varphi - \alpha + \alpha ^{\prime}{)}} \hfill \\ {y_{c} = y_{0} { + }v_{c} t\sin (\varphi - \alpha + \alpha ^{\prime}) - \frac{1}{2}gt^{2} } \hfill \\ \end{array} \hfill \\ y_{c} = y_{0} { + }(x_{c} - x_{0} )\tan (\varphi - \alpha + \alpha ^{\prime}) - \frac{1}{2}g\left( {\frac{{x_{c} - x_{0} }}{{v_{c} \cos (\varphi - \alpha + \alpha ^{\prime})}}} \right)^{2} \hfill \\ \end{gathered} \right.$$

where, (*x*_*0*_*, y*_*0*_) is the initial position coordinates of the soil particles; *g* is the gravity acceleration, 9.8 m/s^2^.

According to formula (8), the trajectory of soil particles thrown out by rotary tiller impact is parabolic, and when the particle throwing angle *φ*-*α* + *α’* is 45°, the distance the particles are thrown out is the farthest. Combined with the actual operating conditions of the developed planter and the corresponding parameter values are substituted, the maximum vertical height and horizontal displacement of the thrown soil can be calculated, which provides a design basis for arranging the seed dropping device and the relative position of the cavity bottom plate and the rotary tiller spindle.

### Analysis of field performance test

The experiments were carried out at the Baima Teaching and Research Base of Nanjing Agricultural University, Lishui District, Nanjing City, Jiangsu Province in November 2020. The first crop of rice straw in the test plot was returned to the field in full amount after the machine harvest, and the variety was Nanjing 9108, which was planted within the full operation width. The measured straw mulch amount was 2.03 kg/m^2^, the moisture content was 19.4%, the average stubble height was > 200 mm, the soil firmness at the depth of 10-15 cm was 18.9Mpa, and the soil moisture content was 19.3%. And the tractor Dongfeng 1404 was used.

To better evaluate the overall operation effect of the developed strip fertilization planter for straw crushing with back-throwing and interrow-laying, the main working parameters that affect the quality of the broken straw strip-paving and the growth of the seed belt crop were selected: the speed of the crushing spindle *A*, the walking speed of the whole machine *B*, the speed of the conveying impeller *C* as the test factors, and the straw mulching uniformity between rows was used as the evaluation index *Y*_*1*_ to characterize the effect of straw mulching, the seed-belt width variability was used as the evaluation index *Y*_*2*_ to characterize the quality of the seed belt, so the field performance tests of the fertilizing planter were carried out. Referring to the similar literature [16, 18, 24] on the operating performance research of straw crushing and returning to the field and no-tillage planter, combined with the preliminary pre-test results of the research group and the actual operation experience basis, the appropriate factors and levels were selected, as shown in Table 2. A 3-factor 3-level orthogonal performance test (*L*_*9*_*(3*^*4*^*)*) was designed to obtain the best overall machine working parameters and operating conditions.

**Table 2:** Factors and levels of orthogonal test

Levels	Factors		
	Crushing spindle speed *A*/(r/min)	Machine walking speed *B*/(m/s)	Conveying impeller speed *C*/(r/min)
1	1800	0.6	190
2	2100	0.8	210
3	2400	1.0	230

**Fig.8:**
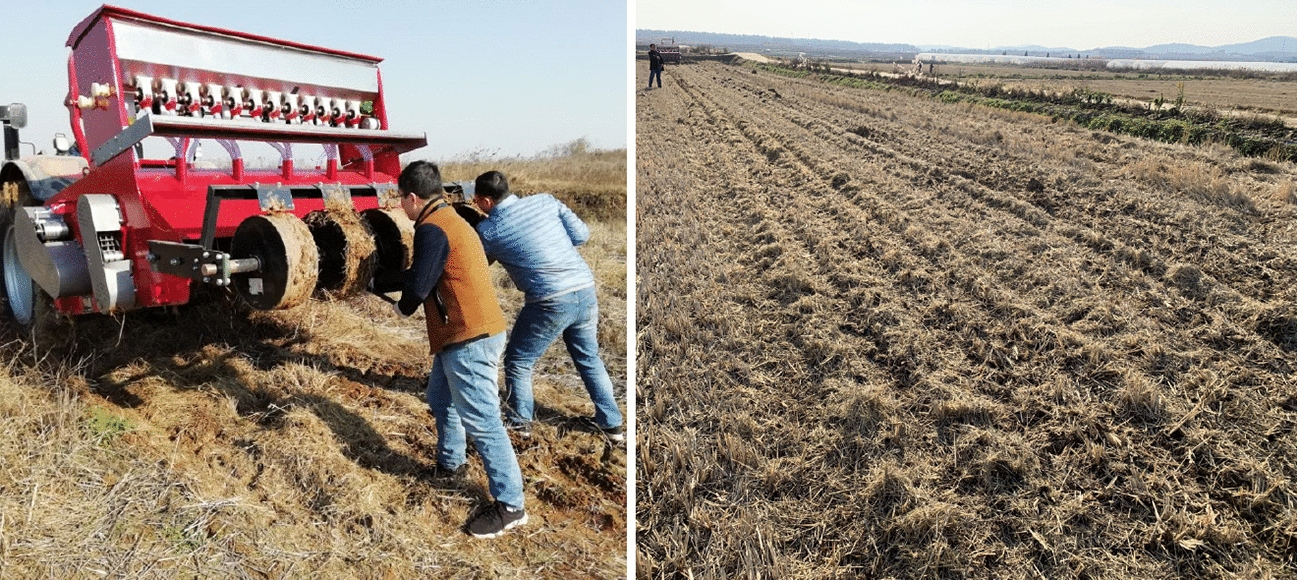
Scene picture of performance test

The test methods and indicators refer to the operation specifications and performance requirements stipulated in the national standard GB/T 20,865–2007 ‘No-tillage fertile-Seeding drill’ [32], GB/T 24,675.6–2009 ‘Conservation tillage equipment-Smashed straw machine’ [33], the agricultural industry standard NY/T 500–2002 ‘Operating quality for crop straw returning-back-to field machine’ [34], and the mechanical industry standard JB/T 8401.3–2001 ‘Smashed root-stubble machine’ [35]. Figure 8 is the field performance test of the whole machine.

During the tests, the corresponding crushing spindle speed *A*, machine walking speed *B*, and conveying impeller speed *C* were adjusted in each single test according to the design scheme. The developed strip fertilization planter was pulled by the tractor, after waiting for the planter calibration and the stable working conditions, the uniformity coefficient *Y*_*1*_ of the straw mulching between rows and the variation coefficient *Y*_*2*_ of the seed-belt width under each parameter combination condition were measured, respectively, to analyze and evaluate the effect of back-throwing and interrow-laying of the strip fertilization planter. After a single test, 10 collection points with an area of 100 mm × 100 mm (a total of 60 collection points) were randomly selected at equal intervals on a diagonal line in each row of the 6 straw mulching areas within the effective working width (2.2 m), and the broken straw mass *w*_*i*_ of each collection point was weighed successively. Similarly, 10 collection points (50 collection points in total) were randomly selected from 5 clean-zone seed belts within the effective working width (2.2 m), and a tape measure was used to measure seed belt width *W*_*j*_ of each collection point successively. Each group of experiments was repeated 3 times and the average value was taken, the calculation formulas of the corresponding test evaluation indexes for the uniformity of straw mulching between rows *Y*_*1*_ and the variability of seed-belt width *Y*_*2*_ were calculated as follows:

12 $$\left\{ \begin{gathered} Y_{1} = 100{\text{\% }} - \frac{{\sum\limits_{i = 1}^{{n_{i} }} {\left| {w_{i} - \overline{w} } \right|} }}{{\sum\limits_{i = 1}^{{n_{i} }} {w_{i} } }} \times 100\% \hfill \\ \overline{w} = \frac{1}{{n_{i} }}\sum\limits_{i = 1}^{{n_{i} }} {w_{i} } \, \left( {i = 1,2,3,...,n_{i} } \right) \hfill \\ \end{gathered} \right.$$

13 $$\left\{ \begin{gathered} Y_{2} = \frac{{S_{W} }}{{\overline{W} }} \times 100\% \hfill \\ S_{W} = \left\{ {\sum\limits_{j = 1}^{{n_{j} }} {[(W_{j} - \overline{W} )^{2} ]} /(n_{j} - 1)} \right\}^{\frac{1}{2}} \hfill \\ \overline{W} = \frac{1}{{n_{j} }}\sum\limits_{j = 1}^{{n_{j} }} {W_{j} } \, \left( {j = 1,2,3,...,n_{j} } \right) \hfill \\ \end{gathered} \right.$$

where, *Y*_*1*_ is the uniformity of the straw mulching between rows, calculated with reference to Christianson uniformity coefficient [24], %; *Y*_*2*_ is the variability of the seed-belt width, %; *w*_*i*_ is the broken-straw mass in test point *i*, g; $\overline{w}$ is the average value of the broken-straw mass at each test point in a single test, g; *W*_*j*_ is the seed-belt width of clean area in test point *j*, mm; $\overline{W}$ is the average value of the seed-belt width at each test point in a single test, mm; *S*_*W*_ is the standard deviation of the seed-belt width at the test point, mm.

## Results

According to the above-mentioned test method and design scheme, the results of the orthogonal performance test of the developed planter were shown in Table 3, and the range analysis and variance analysis of the test results were conducted (shown in Table 4).

**Table 3:** Results of orthogonal test

Test		Test factors			Uniformity of the straw mulching *Y*_*1*_/%	Variability of the seed-belt width *Y*_*2*_/%
		*A*	*B*	*C*		
1		3	3	1	89.72	14.36
2		1	2	3	87.61	12.51
3		3	1	3	91.06	15.24
4		1	3	2	86.33	9.44
5		2	3	3	85.14	14.93
6		3	2	2	92.46	8.95
7		2	2	1	90.67	13.07
8		2	1	2	86.48	10.72
9		1	1	1	84.75	16.08
Uniformity of the straw mulching *Y*_*1*_	*k*_*1*_	86.23	87.43	88.38	*A* > *B* > *C*	*A*_*3*_*B*_*2*_*C*_*2*_
	*k*_*2*_	87.43	90.25	88.42		
	*k*_*3*_	91.08	87.06	87.94		
	*R*	4.85	3.19	0.48		
Variability of the seed-belt width *Y*_*2*_	*k*_*1*_	12.67	14.01	14.50	*C* > *B* > *A*	*A*_*1*_*B*_*2*_*C*_*2*_
	*k*_*2*_	12.91	11.51	9.70		
	*k*_*3*_	12.85	12.91	14.23		
	*R*	0.24	2.50	4.80

**Table 4:** Analysis of variance

Evaluation index	Variation source	Sum of squares	Degree of freedom	Mean sum of square	*F*	Significance
Uniformity of the straw mulching *Y*_*1*_	Calibration model	56.92^a^	6	9.48	3.23	
	*A*	38.28	2	19.14	6.52	**
	*B*	18.20	2	9.10	3.10	*
	*C*	0.43	2	0.21	0.07	
Variability of the seed-belt width *Y*_*2*_	Calibration model	53.11^b^	6	8.85	15.13	
	*A*	0.08	2	0.04	0.07	
	*B*	9.44	2	4.72	8.07	*
	*C*	43.57	2	21.78	37.25	**

‘**’ means extremely significant (significance level < 0.01), ‘*’ means significant (significance level < 0.05)

^a^*R*^*2*^ = 0.91

^b^*R*^*2*^ = 0.98

From the numerical analysis of the range R of various factors in Table 3, it can be seen that for different evaluation indexes *Y*_*1*_ and *Y*_*2*_, the effects of the test factor *A*, *B*, and *C* are significantly different. For the evaluation index *Y*_*1*_, the significance order of the factors is *A* > *B* > *C*, indicating that the speed of the crushing spindle has the greatest influence on the uniformity of straw mulching *Y*_*1*_, followed by the walking speed of the whole machine, the speed of the conveying impeller is the least, and the optimal factor-level combination is *A*_*3*_*B*_*2*_*C*_*2*_. For the evaluation index *Y*_*2*_, the significance order of each factor is *C* > *B* > *A*, indicating that the speed of the conveying impeller has the greatest influence on the variability of the seed-belt width *Y*_*2*_, the walking speed of the whole machine is the second, the speed of the crushing spindle has the least impact, and the optimal combination of factor levels is *A*_*1*_*B*_*2*_*C*_*2*_.

According to the variance analysis results in Table 4, it can be seen from the variance analysis of evaluation index *Y*_*1*_ that *F*_*A*_ > *F*_*B*_ > *F*_*C*_, indicating that the influence of factor *A* on index *Y*_*1*_ is extremely significant, the influence of factor *B* is significant, and the influence of factor *C* is slight. Through the variance analysis of the evaluation index *Y*_*2*_, it can be seen that there is *F*_*C*_ > *F*_*B*_ > *F*_*A*_, indicating that factor *C* has an extremely significant impact on index *Y*_*2*_, factor *B* has a significant impact, and factor *A* has a minor impact, which is consistent with the range analysis results above-mentioned. Combining the results of range and variance analysis, it can be seen that the influence of various test factors (*A*, *B* and *C*) on different evaluation indexes (*Y*_*1*_, *Y*_*2*_) is different in significance, and the corresponding optimal factor-level combination is also different. When the evaluation result was given priority to index *Y*_*1*_, factors *A* and *B* had significant influence on index *Y*_*1*_, and factor *C* had no significant influence, while factor *C* had an extremely significant influence on index *Y*_*2*_, and the factor-level combination *A*_*3*_*B*_*2*_*C*_*2*_ was the best. When index *Y*_*2*_ was given priority, factor *C* and *B* had significant influence on index *Y*_*2*_, factor *A* had no significant influence, while factor *A* had an extremely significant influence on index *Y*_*1*_, and the factor-level combination *A*_*1*_*B*_*2*_*C*_*2*_ was the best.

## Discussion

Further analysis showed that the evaluation index *Y*_*1*_ increased with the increase of the test factor *A*, indicating that the uniformity of the inter-row straw mulching could be improved by increasing the rotating speed of the crushing spindle. The higher the speed of the crushing spindle, the better the straw crushing effect, and the greater the uniformity coefficient of the broken-straw inter-row paving. However, the excessive crushing spindle speed will bring unnecessary power consumption, so it is necessary to balance the appropriate crushing spindle speed to match the power requirements of the whole planter on the basis of meeting the requirements of straw crushing and straw mulching uniformity. The index *Y*_*1*_ showed a trend of first increasing and then decreasing with the increase of factor *B* (which was parabolic shape), and the peak value appeared at *B*_*2*_, indicating that too small or too large machine walking speed will cause *Y*_*1*_ to increase. his is because the slower the machine travels, the more likely it is that the smashed straw will be intermittent during the strip paving process, which leads to an increase in the uniformity coefficient of the mulching straw per unit area. However, the larger the walking speed, the easier the broken-straw to be blocked at the diversion point of the regulating control device in a limited time, which is not conducive to the smooth sliding of the broken straw to both sides, and it will also increase the uniformity coefficient of the straw mulching. Factor *C* has no significant influence on index *Y*_*1*_, so detailed analysis will not be made here.

The evaluation index *Y*_*2*_ presented a trend of first decreasing and then increasing with the increase of test factor *C* (which was concave parabola), the peak value appeared at *C*_*2*_, indicating that too small or too large conveying impeller speed will cause *Y*_*2*_ to increase. Because the impeller speed is too slow, a large amount of broken straw picked up and crushed by the straw crushing device is concentrated at the feed inlet of the conveying impeller, causing a lot of clogging of the broken straw. However, if the impeller rotating speed is too high, the conveying impeller will concentrate a large amount of broken straw at the deflector plate of the diversion control device, which will not be able to smoothly diverging and strip-paving in a short period of time, and the sparse and scattered laying of broken-straw will increase the variation coefficient *Y*_*2*_ of the seed-belt width. The index *Y*_*2*_ is also presented a trend of first decreasing and then increasing with the increase of factor *B*, the peak value appeared in *B*_*2*_, indicating that appropriately increasing the planter walking speed within a certain range is beneficial to the effect of broken-straw distribution and laying, and improve the variability of the seed belt width. While when the walking speed exceeds a certain value, the broken-straw that is going to be diverging and strip-paving cannot slip off in a fast and short time, and will be scraped or pushed by the regulating control device to a certain extent, destroying the ridge shape of straw mulching between rows, resulting in a gradual increase in the variation coefficient of the seed belt width. Similarly, the influence of factor A on the index *Y*_*2*_ is not significant, and no detailed analysis will be made here.

Therefore, combining the evaluation index *Y*_*1*_ (the uniformity coefficient of the straw mulching between rows) and *Y*_*2*_ (the variation coefficient of the seed-belt width), it can be found that the best level of factor *A* that affects index *Y*_*1*_ the most significant is *A*_*3*_, and the best level of factor *C* that affects index *Y*_*2*_ the most significant is *C*_*2*_, and the optimal factor-level combination is *A*_*3*_*B*_*2*_*C*_*2*_. Considering from the perspective of the machine power consumption and the clean energy, under the condition of satisfying the straw crushing effect, ensuring certain straw mulching uniformity and seed-belt width variability, it needs to minimize the power consumption and reduce the rotation speed of the crushing spindle, there is a relatively optimal combination of factor-level as *A*_*2*_*B*_*2*_*C*_*2*_, which did not appear in the orthogonal test scheme. Therefore, a corresponding field verification test is designed to evaluate the broken-straw strip-paving quality and machine operation effect under the two factor-level combination.

### Test verification and analysis

**Fig.9:**
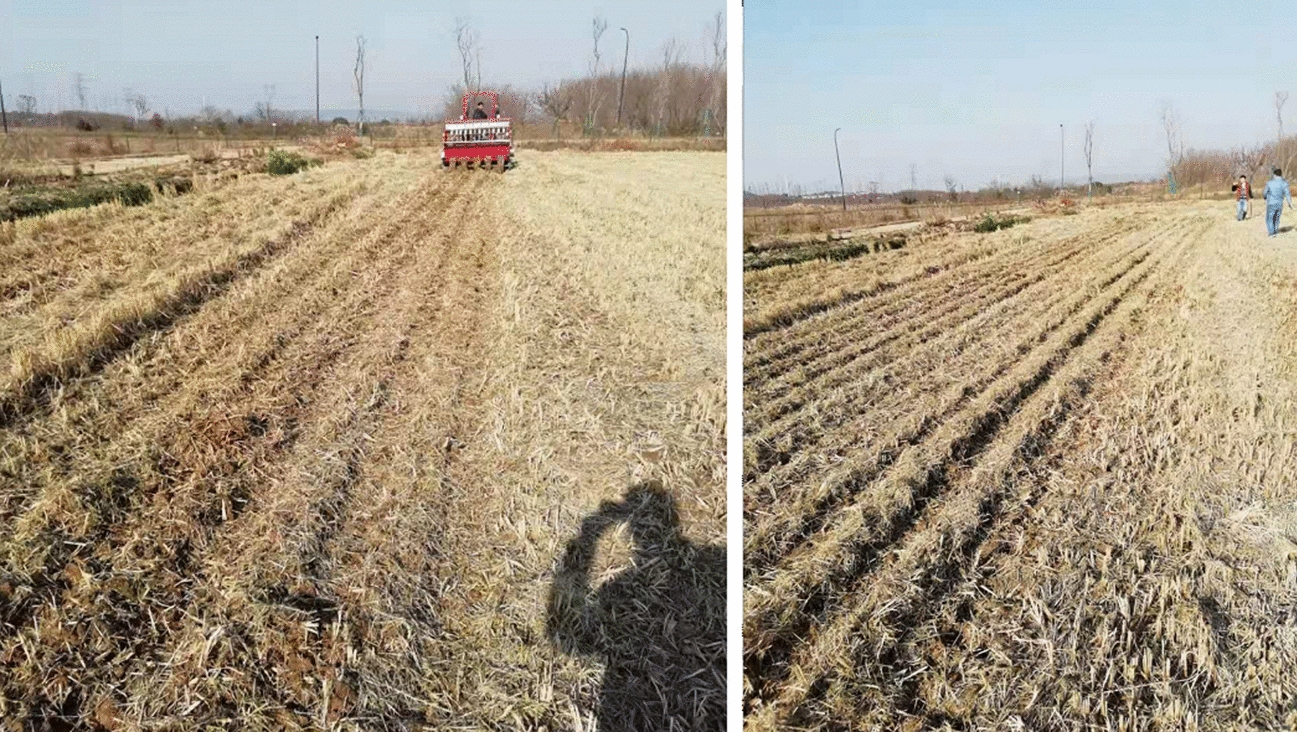
Picture of field validation test and operation effect

In order to verify the rationality of the optimal factor-level combinations obtained by the above orthogonal performance test, a field verification test of no-tillage sowing wheat in the rice stubble field was carried out. Before a single test, the operation parameters of the whole machine were adjusted to the best and better factor-level combinations *A*_*3*_*B*_*2*_*C*_*2*_ and *A*_*2*_*B*_*2*_*C*_*2*_. The test method was consistent with that in Sect. 3.3, and the uniformity coefficient *Y*_*1*_ of the inter-row straw mulching and the variation coefficient *Y*_*2*_ of the seed-belt width under each factor-level combination were determined respectively. Each test group was repeated 3 times and the average value was taken, and a total of 6 repetitive tests were conducted in each group. The field test scenarios and effects were shown in Fig. 9, and the test results were shown in Table 5.

**Table 5:** Results of field test

Test	*A*_*3*_*B*_*2*_*C*_*2*_		*A*_*2*_*B*_*2*_*C*_*2*_	
	Uniformity of the straw mulching *Y*_*1*_	Variability of the seed-belt width *Y*_*2*_	Uniformity of the straw mulching *Y*_*1*_	Variability of the seed-belt width *Y*_*2*_
1	91.72	8.64	88.64	10.86
2	93.51	9.52	93.15	9.24
3	92.49	10.37	92.42	10.68
4	91.22	9.06	90.83	12.38
5	89.34	11.25	89.76	9.72
6	94.06	9.17	90.28	11.51
Average	92.06	9.66	90.85	10.73

Combined with the field operation effect and the verification test results, the developed strip fertilization planter for straw crushing with back-throwing and interrow-laying had good passability and stability, and the full mass straw in the rice stubble field was completely crushed and laid in a regular manner on both sides of the diversion device. For the factor-level combination *A*_*3*_*B*_*2*_*C*_*2*_, the average uniformity coefficient of the straw mulching *Y*_*1*_ was 92.06%, and the average variation coefficient of the seed belt width *Y*_*2*_ was 9.66%. For the factor-level combination *A*_*2*_*B*_*2*_*C*_*2*_, the average uniformity coefficient of the straw mulching *Y*_*1*_ was 90.85%, and the average variation coefficient of the seed belt width *Y*_*2*_ was 10.73%. All the working quality could meet the technical standards of the relevant agricultural machinery industry technical standards and the local agronomic production requirements. Therefore, for the consideration of the machine power consumption and long-term economic cost, the optimal factor-level combination of the operation parameters *A*_*2*_*B*_*2*_*C*_*2*_ was selected, that is, the rotation speed of the crushing spindle was 2100 r/min, the walking speed of the whole machine was 0.8 m/s, and the rotation speed of the conveying impeller was 210 r/min. It can effectively improve the operating performance of the developed strip fertilization planter for straw crushing with back-throwing and interrow-laying, the uniformity of the broken straw inter-row mulching is better, and the variability of the seed-belt width is minor, which provides technical reference for realizing the high quality and smooth no-tillage sowing compound operation in the clean area.

## Conclusions

This study continued the idea of strip fertilization and planting in ‘clean area’, an innovation of strip fertilization planting for straw crushing with back-throwing and interrow-laying was developed, the main factors that affect the uniformity of the broken straw mulching between rows and the variation of the seed-belt width were theoretically analyzed, and the optimal combination of operating parameters was determined to give play to the best operating performance.

The performance test results showed that through the range and variance analysis, it is clear that the significance of the evaluation indexes *Y*_*1*_ and *Y*_*2*_ were in the order of *A*, *B*, *C* and *C*, *A*, *B*, respectively, and the optimal factor-level combination scheme was *A*_*3*_*B*_*2*_*C*_*2*_. Comprehensively considering the power consumption and environmental protection effects, the better factor-level combination scheme was *A*_*2*_*B*_*2*_*C*_*2*_, on the basis of ensuring operation requirements and agronomic requirements.

The field verification test results showed that under the optimized operating parameter combination, the developed strip fertilization planter had good passability and high stability, and the factor-level combination *A*_*2*_*B*_*2*_*C*_*2*_ could save a certain amount of power consumption while meeting relevant industry technical standards and production quality requirements. When the rotation speed of the crushing spindle was 2100 r/min, the walking speed of the whole machine was 0.8 m/s, and the rotation speed of the conveying impeller was 210 r/min, the average uniformity coefficient of the straw mulching *Y*_*1*_ was 90.85%, and the average variation coefficient of the seed belt width *Y*_*2*_ was 10.73%, which achieved better strip-paving quality of broken straw and arrangement effect of seed bed.

## Data Availability

The datasets supporting the conclusions of this article are included within the article and its additional files.
